# The Socket-Shield Technique: Digital Planning, Guided Surgery, and Immediate Implant Loading—2-Year Follow-Up

**DOI:** 10.1155/2022/8188905

**Published:** 2022-11-21

**Authors:** Sušić Mato, Smojver Igor, Stojić Luka, Katalinić Ivan, Gerbl Dražena, Vuletić Marko

**Affiliations:** ^1^School of Dental Medicine, Department of Oral Surgery, Zagreb, Croatia; ^2^St. Catherine Specialty Hospital, Zagreb, Croatia; ^3^Private Dental Office, Zagreb, Croatia; ^4^Department of Anaesthesiology & Critical Care, University Hospital Centre Zagreb, Zagreb, Croatia

## Abstract

Anterior aesthetic zone implant placement and tooth restoration can be a quite challenging procedure. Modern technology offers new tools that can help maximize results in both aesthetic and functional ways. The socket-shield technique, as described by Hürzeler et al., boosted with the 3D diagnostics, digital planning, and 3D printing, could provide a valuable alternative to traditional approaches. This case report describes a clinical workflow for an efficient anterior implant-prosthetic restoration.

## 1. Introduction

Restoring lost frontal tooth can be a complex challenge for every clinician. After the tooth extraction, alveolar bone and adjacent soft tissues start immediate adaptation and remodelation. The labial bone of the upper anterior teeth resorbs relatively fast due to the loss of blood supply from the cancellous bone [1, 2]. This chain of events can impair final aesthetic results of the implant-supported restoration in terms of unpredictable long-term soft and hard tissue margins in regard to the dental crown and the implant neck. Salama et al. [3] demonstrated retaining a tooth root is potentially an effective method of soft and hard tissue preservation. Following this finding, Parlar et al. [4] placed an implant into a previously prepared intra-root space without actually extracting the root itself. Unfortunately, the approach failed due to the lack of osseointegration and connective tissue grown into the space between the implant and the root wall. Hürzeler et al. [5] introduced the socket-shield technique in order to overcome previously described problems. Gluckman et al. [6] further refined the treatment and proposed vertical root sectioning along with mesio-distal cutting into oral and vestibular portions. Probably the main challenge with this relatively new technique is an accurate tooth fragment preparation. A full-thickness flap approach would be useful to gain visual control over the preparation but is not recommended in order to help prevent bone resorption emerging from surgical trauma and temporary loss of blood supply [7]. As previously stated, precision is of paramount importance; proper and precise root segment preparation and implant placement dictate the outcome of the treatment [8]. Digital planning and surgical guides could assist overcoming this issue. With the help of digital technology, one can plan the final crown in advance, prepare temporary crown that can be attached immediately post-operative, and 3D print surgical template to ensure precise implant placement.

This clinical report presents the variation of the socket-shield protocol described by Zhang et al. [9], combining the use of digital planning, surgical guide, and free-hand preparation.

## 2. Case Report

A 47-year-old patient was referred to our clinic because of a horizontal fracture of the root of tooth #11 without other medical problems and diseases. In addition to the above, he also wanted to solve the aesthetics of the remaining front upper teeth that had old metal-ceramic crowns (teeth #12, #21, and #22). After the clinical examination and analysis of the cone-beam computed tomography (CBCT) image, an unfavorable horizontal fracture of tooth #11 was confirmed. Two options for prosthetic rehabilitation were offered: (1) a fixed ceramic bridge on the remaining teeth and (2) an implant in position #11 with new individual crowns on the remaining front teeth (#13, #12, #21, #22, and #23). The patient insisted on individual crowns with an implant at position #11. As it was an aesthetic zone, we decided on a socket-shield procedure due to the favorable fracture line and in order to maintain the morphology of the soft tissues in a minimally invasive way. Considering the sensitivity of adequate prosthetic implant positioning in the aesthetic zone and the consequent impact on long-term aesthetic stability, we insisted on a guided surgical implantation procedure. In this way, we used the benefits of both the socket-shield technique in terms of preserving the soft tissue architecture and the guided surgery for prosthetic positioning of the implant and immediate temporary restoration.

### 2.1. Digital Implant Procedure Planning

**Figure 1:**
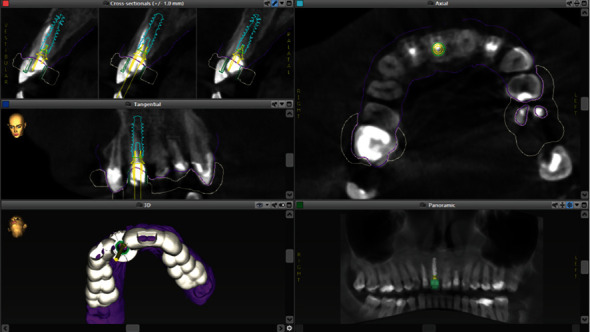
The virtual planning in the specialist software.

**Figure 2:**
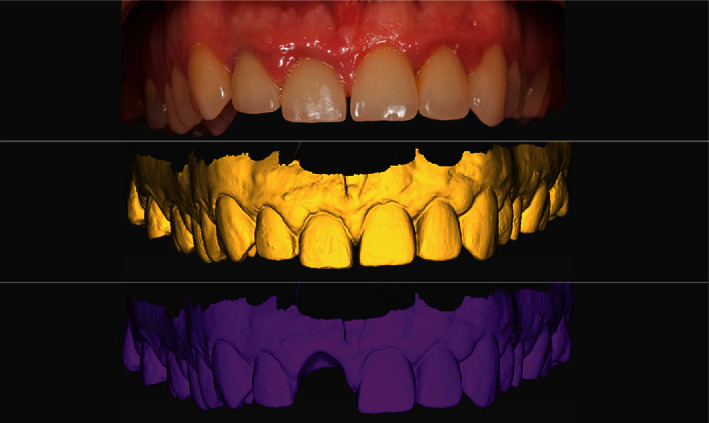
The planning phases overlapped: intraoral starting point—.STL scan of starting position—tooth #11 virtual extraction.

**Figure 3:**
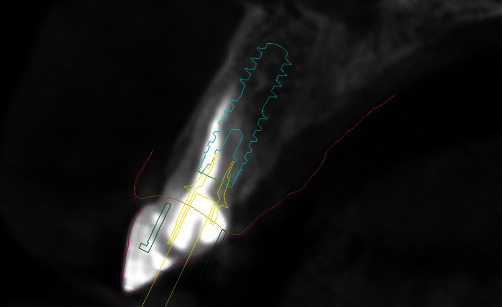
Virtual implant placement planning—superimposition of future implant and the root.

In order to properly prepare for implant placement procedure, the coDiagnostiX® (Dental Wings Inc., Montreal, QC, Canada) digital planning software was used (Figure 1). In this software, the CBCT scan exported in DICOM format (Orthophos SL, Densply Sirona, Bensheim, Germany) was overlapped with the .STL file obtained from patient intraoral scanning with the 3Shape TRIOS 3 scanner (3Shape, Copenhagen, Denmark; Figures 2 and 3).

**Figure 4:**
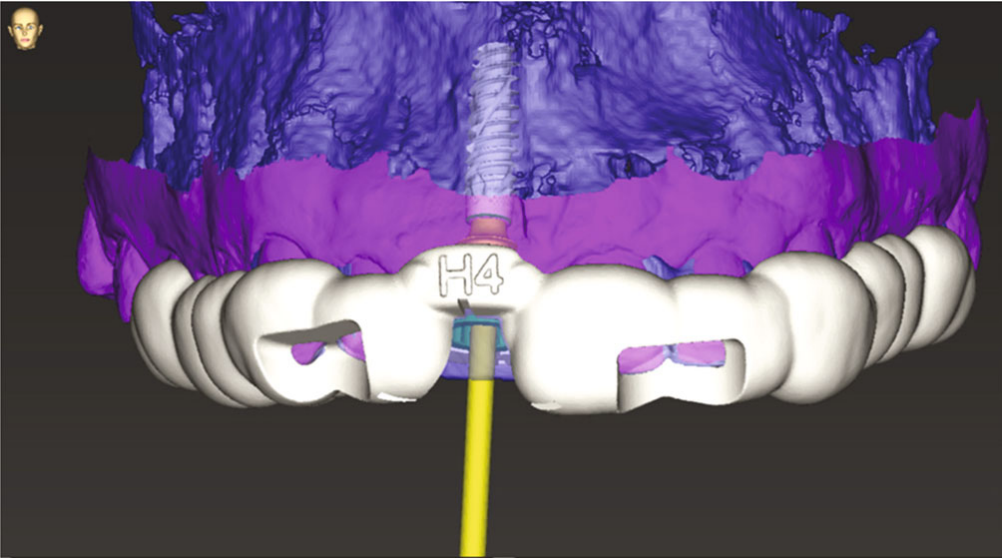
Surgical implant stent digital planning.

After digital merging of DICOM and .STL files, tooth #11 was virtually extracted, and the implant was positioned bearing in mind the final position of the new crown as well as the root fragment that is to remain inside the alveola after the *in vivo* extraction. Surgical stent adjusted for Straumann BLX SLActive RB implant 4.0 length 14 mm (Straumann Group, Basel, Switzerland) was 3D printed following the virtual design (Figure 4).

### 2.2. Dental Laboratory Phase

The future implant position has been digitally planned as follows: virtual model export option together with the “Scanbody” implant position has been selected in the CoDiagnostiX planning software (Straumann CARES RB/WB Mono Scanbody, Straumann Group). The (CAM) computer-aided manufacturing design software (3Shape Dental Designer) has been used for temporary crow design. Working model with prepared space for RB Repositionable Implant Analog has been designed with the 3Shape Model Bilder software. The model has been printed with Straumann® P20+ printer using P Pro Master Model Grey/P Pro Gingiva Mask resin (Straumann Group). The temporary crown (Telio CAD LT, Ivoclar Vivadent, Schaan, Liechtenstein) has been made utilising computer-aided design(CAD)/CAM technology. The crown has been cemented on the implant abutment (RB Variobase®, Straumann Group, for Crown AS) with the self-adhesive composite cement (Multilink® Hybrid Abutment, Ivoclar Vivadent). Surgical guide has been 3D printed (Straumann® P20+, Straumann Group) in Pro Surgical Guide resin (Rapid Shape, Heimsheim, Germany).

### 2.3. Surgical and Restorative Phase

**Figure 5:**
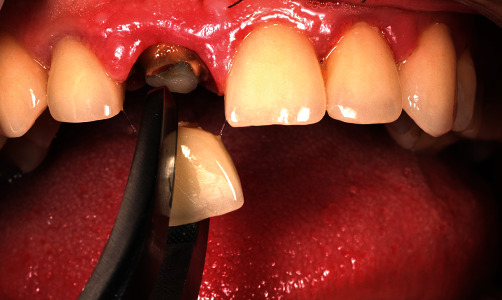
The old crown removal and root preparation/sectioning.

After the application of local anaesthesia (4% articaine with epinephrine 1 : 200,000; 1.8 mL), according to the Zuhr/Hürzeler protocol [5], the fractured clinical crown of the tooth was removed, and the coronal buccal root segment was separated from the rest of the root using a Lindemann surgery bur (NTI, Kahla, Germany) with the visual aid of light and magnification loupes (Figure 5).

**Figure 6:**
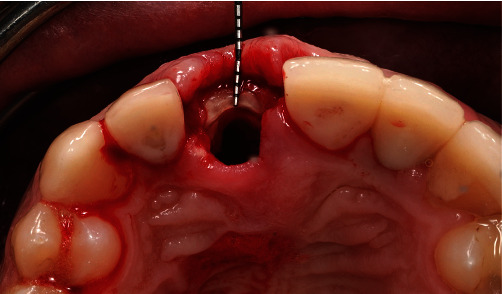
The buccal root “shield” in place after sectioning.

**Figure 7:**
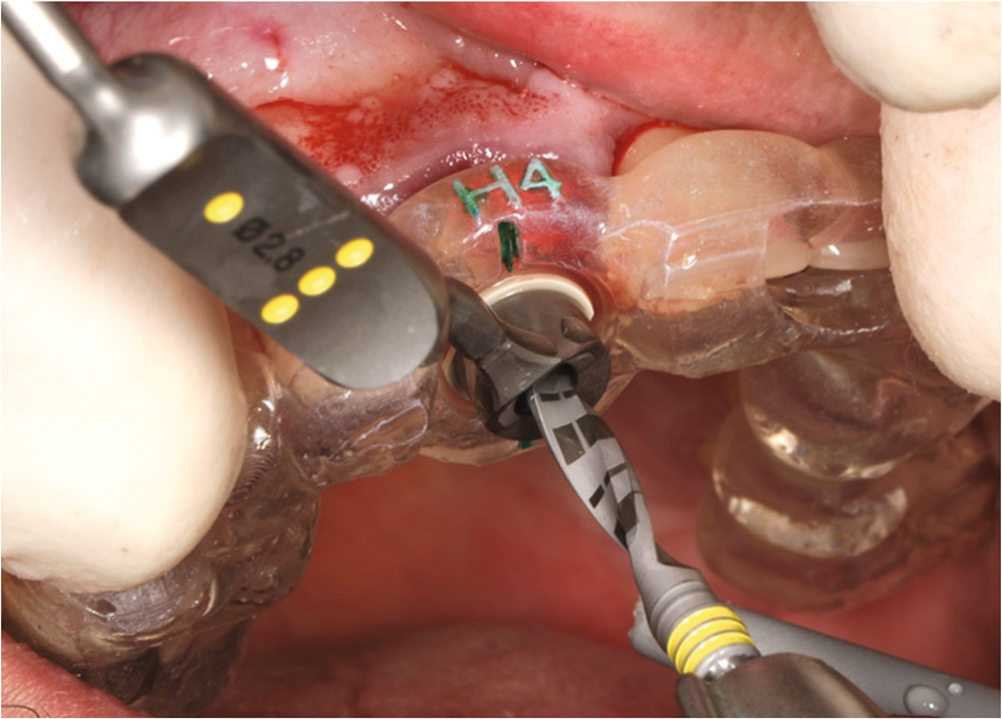
The surgical stent–drill guide fixed in place.

**Figure 8:**
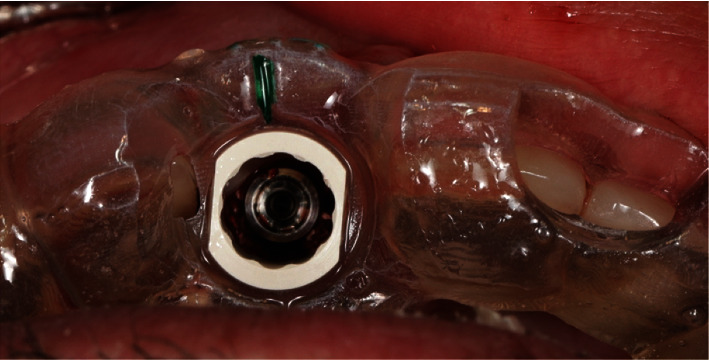
The occlusal view through stent.

**Figure 9:**
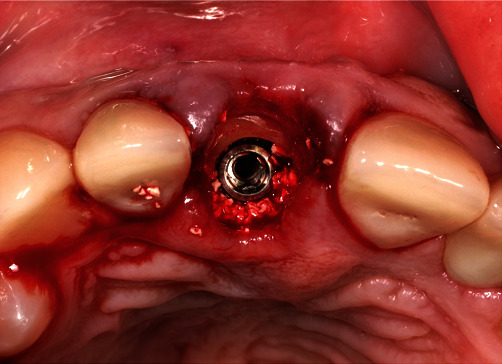
The implant in place—socket shield can be observed buccally along with some xenograft material.

The remaining pieces of the root were removed using a periotome (Carl Martin, Solingen, Germany), without placing excessive stress on the buccal tissues. This resulted in a part of the former root in the area of the buccal bony socket, which was thinned out to a thickness of 2 mm using a round diamond bur (NTI) under sterile saline cooling (Figure 6). Then, implant bed preparation was performed with surgical guide according to the manufacturer's guidelines for BLX full guided surgery (Figures 7 and 8). Before the implant was inserted, a buccal root was smeared with Emdogain gel (Straumann Group) to prevent epithelial proliferation and bacterial colonization [5, 10], and a microgap was filled with xenograft bone substitute (Straumann Xenograft 0.5 g, Straumann Group; Figure 9).

**Figure 10:**
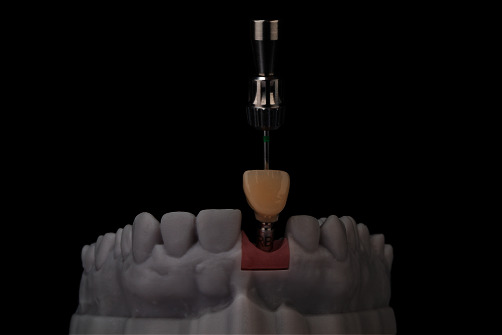
The temporary crown on the 3D printed lab model.

**Figure 11:**
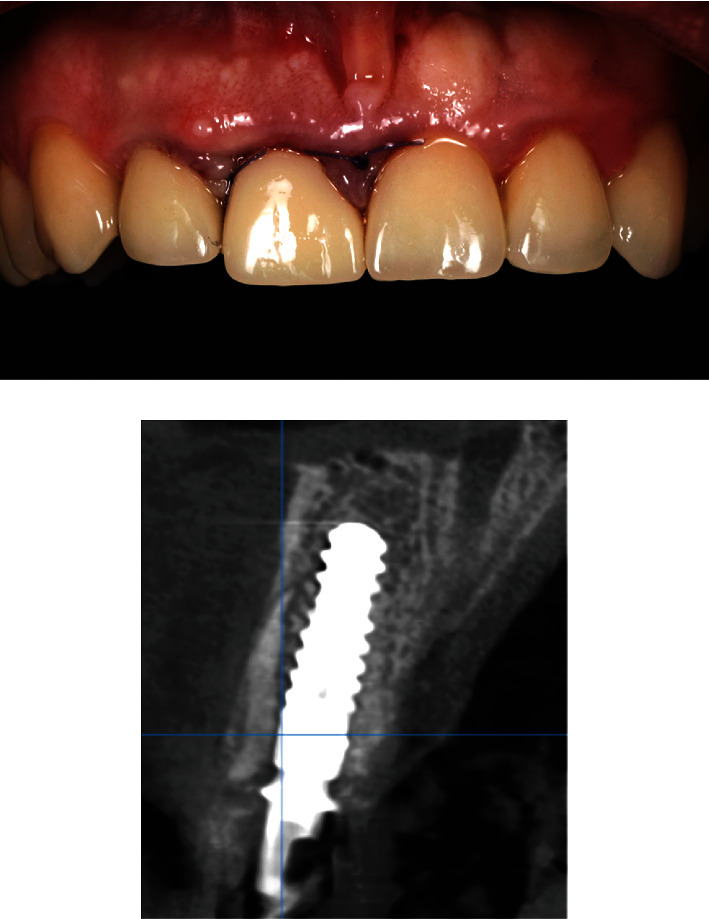
Immediate post-operative with fitted immediate crown and CBCT.

Temporary crown was fitted, and mesial papilla was repositioned with 5–0 monofilament suture (Prolene, Ethicon, Johnson & Johnson, Somerville, NJ, USA; Figures 10 and 11). Antibiotic therapy (amoxicillin and clavulanic acid) was prescribed for 7 days after surgery for all patients.

**Figure 12:**
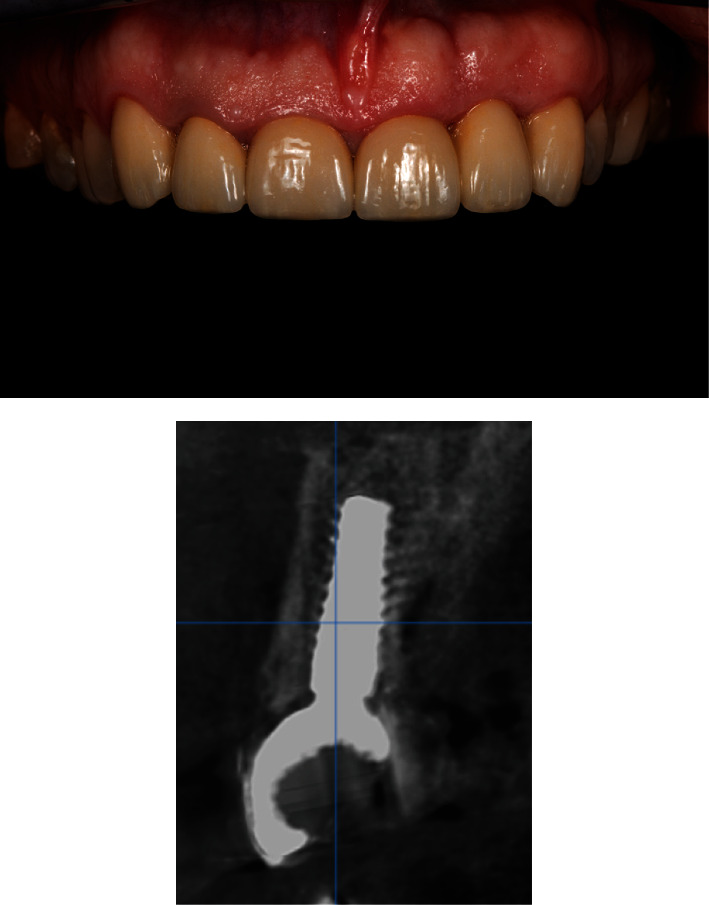
The final restoration in place with control CBCT after 2 years post-operative.

**Figure 13:**
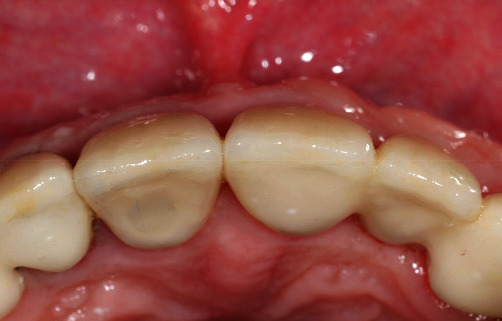
The occlusal view on the tooth #11 and the soft tissue adaptation to the final restoration.

After 6 months, teeth #23, #22, #21, #12, and #23 were prepared and crowned, first with temporary crowns (Protemp 4, 3M, Neuss, Germany) and then with lithium disilicate crowns (IPS e.max ceram, Ivoclar Vivadent). The definitive permanent screw-retained hybrid zirconia ceramic crown was made (Zolid Zirconia block—Amann Girrbach, cladding ceramics Celtra Ceram, Dentsply Sirona, Charlotte, NC, USA). Crown has been cemented on the implant abutment (RB Variobase® for Crown AS) with the self-adhesive composite cement (Multilink® Hybrid Abutment, Ivoclar Vivadent; Figures 12 and 13).

## 3. Discussion

The potential for reducing or avoiding buccal bone loss after the tooth loss has been recorded when opting for conservation of a buccal root portion followed by an immediate implant insertion or so called socket-shield protocol [5]. Preserved root portion retains its periodontal ligament and supra-periosteal attachment, which stabilizes neighboring soft and hard tissues and prevents them from receding [5]. This approach, being a part of a concept called “partial extraction therapies,” challenges the well-established “extract and augment” approach [11]. Although it seems quite promising, some complications may emerge during socket-shield protocol. They are mainly connected with improper management of the root fragment and unprecise implant placement. For instance, if there are pulp tissue remnants, an inflammation can occur. If the root fragment is too thin, socket-shield could fracture. In addition, the large contact surface between an implant and a root can cause root fragment migration and lower rate of osseointegration (connective tissue ingrowth) [12]. Free-hand preparation of the root fragment followed by implantation requires rather skilled clinician and good pre-operative preparation, also keeping final restoration in mind. The 3D printed surgical guide based on 3D planning is a tool that can help in both properly preparing remaining root fragment and assist with precise implant insertion. In contrast to Zhang et al. [9] clinical case report, we did not use two guides: one for the root fragment preparation and other for implantation. Instead, we have used only one template, which is for an implant placement. In the current case, it was more convenient for the surgeon to adequately prepare the root segment free-handed, due to the need for crown lengthening procedure. In order to shape final aesthetics of the future crown and adjust it to the adjacent tooth, the edge of the bone (the anterior socket wall) was reduced by 1 mm. Thus, the edge of the root fragment was also reduced by 1 mm. Sharp new drills, as proposed by Zhang et al. [9], have been used to minimize the damaging potential on the shield and adjacent tissues. We have chosen Straumann BLX SLActive® (Straumann Group) implant for this specific case. The hydrophilic nano-structured implant surface should increase chances for successful osseointegration as this type of surface supports potent fibrin network formation and mineralization [13].

The implant crown shape, size, and position were also planned prior the treatment, which enabled us to provide the patient with the aesthetic temporary restoration immediately after the implantation. This detail improves predictability of the adjacent soft tissue remodelation. Finally, this modified socket-shield protocol allowed us to achieve predictable and stable results: good osseointegration, proper function, and satisfying aesthetics.

## 4. Conclusion

The modified socket-shield technique shown in this case, using both free-hand preparation of the field of interest along with the guided implantation based on previous 3D analysis and planning, showed satisfying results in both functional and aesthetic aspects without facing serious complications during or after the treatment.
